# *Isochrysis maritima* Billard and Gayral Isolated from Penang National Park Coastal Waters as a Potential Microalgae for Aquaculture

**DOI:** 10.21315/tlsr2017.28.2.12

**Published:** 2017-07-31

**Authors:** Mohammad Basri Eshak, Wan Maznah Wan Omar

**Affiliations:** 1 School of Biological Sciences, Universiti Sains Malaysia, 11800 USM Pulau Pinang, Malaysia; 2 Centre for Marine and Coastal Studies (CEMACS), Universiti Sains Malaysia, 11800 USM Pulau Pinang, Malaysia

**Keywords:** *Isochrysis maritima*, Polyunsaturated Fatty Acid, Specific Growth Rate, Aquaculture Feed

## Abstract

The importance of polyunsaturated fatty acid (PUFA) in microalgae was widely reported. In this study, six isolated microalgae from Teluk Aling, Penang National Park were screened for PUFA contents. *Isochrysis maritima* showed the best polyunsaturated fatty acids essential for aquaculture species compared to other microalgal species tested. This species is a good choice as aquaculture feed due to its small size (3–7 μm), which is appropriate size for ingestion. The maximum specific growth rate of this species was also high (0.52–0.82 days^−1^) and comparable with many recognised aquaculture microalgae. On the other hand, this species was also able to be cultivated successfully in big volume (1000 L culture medium) with open hatchery condition, which will optimise the production cost. Low ratio of omega-6 to omega-3 essential fatty acids (EFA) recorded in *I. maritima* at any growth phases (0.32–0.45) also indicate optimal values for feeding.

## Abstract (ru)

Kepentingan asid lemak poli tak tepu (PUFA) dalam mikroalga telah dilaporkan secara meluas. Dalam kajian ini, enam mikroalga yang diasingkan dari Teluk Aling, Taman Negara Pulau Pinang telah disaring untuk kandungan PUFA. *Isochrysis maritima* menunjukkan bacaan asid lemak poli tak tepu yang terbaik yang penting untuk spesies akuakultur berbanding spesis microalga lain yang diuji. Spesis ini adalah pilihan yang baik sebagai pemakanan akuakultur kerana saiz yang kecil (3–7 μm), iaitu saiz yang sesuai untuk pemakanan. Kadar maksimum pertumbuhan spesifik spesis ini juga tinggi (0.52–0.82 hari^−1^) dan setanding dengan kebanyakan mikroalga akuakultur yang telah diiktiraf. Selain itu, spesies ini juga boleh dikultur dengan jayanya dalam skala yang besar (1000 L media kultur) di tempat penetasan yang terbuka, yang akan mengoptimumkan kos pengeluaran. Nisbah omega-6 kepada omega-3 asid lemak (EFA) yang rendah dicatatkan pada *I. maritima* pada mana-mana fasa pertumbuhan (0.32–0.45) juga menunjukkan nilai yang optima sebagai makanan akuakultur.

## INTRODUCTION

Microalgae are widely utilised in aquaculture industry as live food and feed additive in the commercial rearing of many aquaculture species (Mata *et al.* 2010). It is either for direct consumption such as for mollusks and peneid shrimp or indirect consumption as food for the live prey like rotifers and artemia to feed small-larvae fish (Patil *et al.* 2005). The most common utilised microalgae species as feed are *Chlorella*, *Tetraselmis*, *Isochrysis*, *Pavlova*, *Phaeodactylum*, *Chaetoceros*., *Nannochloropsis*, *Skeletonema* and *Thalassiosira* (Spolaore *et al.* 2006; Hemaiswarya *et al.* 2010).

Polyunsaturated fatty acid (PUFA), for example docosahexaenoic acid (DHA), eicosapentaenoic acid (EPA) and arachidonic acid (AA) content is a major importance (Spolaore *et al.* 2006; Hemaiswarya *et al*. 2010). EPA and DHA are essential structural components of cell membranes, with DHA in particular playing an essential functional role in the development of neural and visual cells (Glencross 2009). Eicosanoic acids such as EPA and AA are essential for the production of eicosanoids, which are a wide group of hormones that play important roles in immune and neurological responses, osmoregulation, and controlling the stress response, steroid biosynthesis and smooth muscle contraction in animals (Bransden *et al*. 2005; Glencross 2009). The importance of microalgae as a source of PUFA has been widely reported. The microalgae also may have superior lipid stability compared with traditional PUFA, because they are naturally rich in antioxidant carotenoids and vitamins, and their lipids are bioencapsulated by the cell wall (Patil *et al.* 2005). Therefore, microalgae are important feed sources in aquaculture due to their nutritional value and their ability to synthesize and accumulate great amounts of PUFA.

The microalgae utilised as feed in hatcheries vary in size, environmental requirements, growth rate, and nutritional value (Helm *et al.* 2004). Brown (2002) outlined several key attributes for microalgae to be good aquaculture species. Firstly, they must be in accordant size for ingestion, for example, size range from 1 to 15 μm for filter feeder species and 10 to 100 μm for grazer species. Subsequently, they must possess rapid growth rates, able to grow in mass size cultivation, and also unsusceptible to any fluctuations in temperature, light and nutrients which may occur in hatchery systems. Eventually, they must possess a good nutrient composition such as protein, carbohydrate, lipid and fatty acids, plus lack of toxins which might be transferred up through food chain.

Marine microalgae are frequently used as food sources in cultivation of marine herbivores, and efforts have been made to study the lipid content and fatty acid composition of different algae used as food for bivalves, zooplankton such as rotifers and artemia (Reitan *et al.* 1994), and the larval stages of crustaceans and fishes (Brown *et al.* 1997; Muller-Feuga 2004; Guedes & Malcata 2012). Many marine fish larvae have high dietary requirements for essential omega-3 highly unsaturated fatty acids for normal growth and development (Reitan *et al.* 1994). Therefore, our aim was to isolate new strain that is capable of producing essential fatty acids and possess other attributes suitable for aquaculture from Penang coastal waters. The objectives of the present study were twofold: (1) to screen the microalgae isolated from the Penang coastal waters for PUFA (LA, ALA, DHA, EPA & AA) and (2) to investigate the growth performance and biochemical compositions of selected species in series of batch culture.

## MATERIALS AND METHODS

### Isolation of Microalgal Strain

Microalgal strains were isolated from the samples collected at the area surrounding the Penang National Park coastal waters. The samples collected were inoculated in sterilised Walne’s media (Andersen *et al.* 2005) for 2 weeks before the isolation process begin with no vitamins added to ensure that the microalgae strains obtained can grow photoautotrophically and produce high amount of biochemical compounds especially polyunsaturated fatty acid (PUFA) without vitamin requirement. After two weeks, the samples were centrifuged (Table top Centrifuge M4000, Kubota, Japan) at 3000 xg for 10 min. The supernatant was discarded and replenished with new media. The samples were centrifuged again for a few times with the same speed and time to reduce the amount of contaminants in the culture. To isolate the microalgae, 50 μL of the culture samples were inoculated on the same media solidified with 1.5% of agar. Samples were incubated on algae shelve inside algae culture room at 25 ± 1.0°C under 50 μmol m^−2^s^−1^ of white fluorescent light with 24 h light duration. The colonies formed were sub-cultured at least three times on fresh agar plates to ensure that all microalgae were totally isolated into single species. All microalgae species isolated were sent to Biotech International R & D (BIRD) Centre, Egypt for identification.

### Preliminary Screening on Polyunsaturated Fatty Acid (PUFA)

50 mL of all species isolated namely *Isochrysis maritima*, *Isochrysis galbana*, *Chaetoceros calcitrans*, *Tetraselmis tetrathele*, *Chlorella sorokiniana* and *Nannochloropsis oculata* were inoculated into 200 mL sterilised Walne’s media. The media was sterilised by autoclaving at 121°C at 15 psi above atmospheric pressure for 15 min. The initial cell density was adjusted to 2.5 × 10^5^ cell.mL^−1^ after introduction into the culture media. All cultures were incubated on algae shelve inside algae culture room at 25 ± 1.0°C under 50 μmol m^−2^s^−1^ of white fluorescent light with light duration 12 : 12 h light: dark cycle. Agitation of the culture medium was conducted by shaking the flasks twice daily. All the cultures were harvested by centrifugation (High Capacity Tabletop Centrifuge M8420, Kubota, Japan) at 6000 xg for 10 min after it reach early stationary phase. The cell pellets were freeze-dried for PUFA contents measurement. All cultures were extracted by direct transesterification method as reported by Abel *et al.* (1963) with some modification and analysed by gas chromatography (GCMS-2010, Shimadzu, Japan). Microalgae which possess the best PUFA contents were further examined to check the suitability of that species for aquaculture.

### Growth Rate Measurement of Microalgal Isolates

*Isochrysis maritima* was cultivated triplicate under batch conditions in 0.1 L, 2 L, 10 L and 1000 L Walne’s media. The experiments were started by inoculating stock solution of *I. maritima* at exponential phase into 250 mL conical flasks containing 0.1 L of sterilised Walne’s media. The media was sterilised by autoclaving at 121°C at 15 psi above atmospheric pressure for 15 min. The initial cell density was adjusted to 2.5 × 10^5^ cell.mL^−1^ after introduction into the culture media. The culture was maintained in algae culture room at 25 ± 1.0°C under 50 μmol m^−2^s^−1^ white fluorescent light with light duration 12 : 12 h light: dark cycle for 14 days. Agitation was conducted by shaking the flasks twice daily. The cell density was counted everyday by using haemacytometer (Neubauer-Improved Haemacytometer, Labor Optik, United Kingdom). Then, a time versus cell density growth curve was plotted.

This study was continued with the same procedures of inoculation for 2 L, 10 L and 1000 L Walne’s media. The Walne’s media used was sterilised by sodium hypochlorite 5.25 %(w/v) overnight with the ratio of 1 mL sodium hypochlorite to 1 L seawater and neutralised by sodium thiosulphate (250 g.L^−1^) with the ratio of 1 mL sodium thiosulphate to 4 L sodium hypochlorite (Kawachi & Noël 2005). *I. maritima* in 2 L Walne’s media was incubated in the same condition as in 0.1 L Walne’s media with aeration at the rate of 1 L min^−1^, whereas for 10 L and 1000 L Walne’s media, they were incubated in open hatchery for 14 days with uncontrolled environmental conditions (temperature range: 25°C–31°C; light range: 25–1000 μmol m^−2^s^−1^; light duration: 12: 12 h light: dark cycle). Agitation for 10 L and 1000 L Walne’s media were provided by continuously bubbled with 0.2 μm filtered air at a rate of 1 L min^−1^.

The specific growth rate was determined by plotting the natural logarithm of culture cell density against time. Readings within the exponential phase were then used to obtain the maximum specific growth rate by linear regression (Pahl *et al.* 2010). The specific growth rate was calculated according to this equation (Alkhamis & Qin 2013):

$$\mu =\frac{\left(\text{ln }X_{2}-\text{ln }X_{1}\right)}{\left(t_{2}-t_{1}\right)}$$

where *X*_2_ and *X*_1_ are the cell density (cell.mL^−1^) at time *t*_2_ and *t*_1_ (day), respectively.

### Biochemical Compositions of Microalgal Isolates

This experiment was carried out triplicate in 10 L Walne’s media separately from growth rate study. The seawater was sterilised using sodium hypochlorite 5.25%(w/v) overnight and neutralised by sodium thiosulphate (250 g.L^−1^) (Kawachi & Noël 2005). The initial density was adjusted to 2.5 × 10^5^ cell.mL^−1^ after introducing into Walne’s media (Fidalgo *et al.* 1998). The culture were grown at hatchery (temperature range: 25°C–31°C) under white fluorescent light (50 μmol m^−2^s^−1^) for 12: 12 h light: dark cycle and aeration with 0.2 μm filtered air at a rate of 1 L min^−1^. The number of cells was counted daily for 14 days. Then, the microalgae were harvested at three different growth stages including exponential (day 5), early stationary (day 7) and late stationary (day 10) stages by flocculation method (Şirin *et al.* 2011). The microalgae were then analysed for total protein, total carbohydrate and fatty acids composition.

### Analytical Methods

The cell density was counted everyday by using haemacytometer (Neubauer-Improved Haemacytometer, Labor Optik, United Kingdom). Cell biomass was determined by dry weight and ash content at the end of experiment (Van Wychen & Laurens, 2013). Samples were harvested by flocculation at the same time for all growth phases by adjusting the pH to 10–11 using 1M NaOH. The flocculated cells were centrifuged (Tabletop Centrifuge M4000, Kubota, Japan) at 3000 xg for 5 min. The pellets were washed with distilled water and were centrifuged again at the same speed and time. The cell pellets were freeze-dried for total protein, total carbohydrate and fatty acid content measurement. Protein content was extracted using a method proposed by Rausch (1981) and measured by Lowry assay (Lowry *et al.* 1951). Carbohydrate was extracted using a method proposed by Chu *et al. (*1996) and measured using phenol-sulphuric assay by the method of DuBois *et al. (*1956).

### Fatty Acid Analysis

Microalgal cells were extracted by direct transesterification method as reported by Abel *et al.* (1963) with some modification. Dried cells of 100 mg were weighed and transferred into screw cap bottles, and 2 mL of methanolic sulphuric acid (15% v/v) was added into the screw cap bottles together with 2 mL of chloroform. Then, the mixture was bubbled with nitrogen gas for 15 seconds and subsequently vortexed (Reax 2000, Heidolph, Germany) for 2 min. After visibly homogenous, the mixture was heated using heater block (HB-48, Wisetherm, Germany) at 80°C for 30 min. Then, 1 mL of distilled water was added and was vortexed again for 30 seconds. Two layers of solution were formed. Afterwards, the lower layer of the mixture was transferred into 2 mL vial and centrifuged at 10000 xg for 5 min. Ten milligram of sodium sulphate anhydrous was added to make sure all water was completely removed from the mixture. Finally, 0.75 μL of the lower layer was transferred into the gas chromatography (GC) vial. It was sealed and kept at −20°C until GC analysis was carried out.

Fatty acid methyl esters (FAME) were separated and quantified by gas chromatography (GCMS-2010, Shimadzu) equipped with a flame ionization detector and a 30 mm × 0.22 mm 70% cyanocropylpolysilphenylene–siloxane (BPX70, SGE). Nitrogen was used as carrier gas and temperature programming was set from 100°C to 210°C at 2°C/min, and then maintained at 210°C for 30 minutes. The injector and detector temperature were set at 250°C and 260°C, respectively. Menhaden oil and Supelco 37 Component FAME Mix were used as a standard for fatty acid identification by comparison of peak retention times between samples and standards with the ratio of 3:1. The concentration of the fatty acids were estimated from the peak area on the chromatogram using methyl enanthate 99% (C7:0) (Sigma Aldrich, USA) as an internal standard.

### Statistical Analysis

Data were treated statistically by one-way analysis of variance (ANOVA) using SPSS (Statistical Package for the Social Sciences) V20.0 software to test for possible significant differences in means of dependent variables among the strains tested, and the different existed was determined by Duncan Test. All experiments were done in triplicate and all data was presented in mean ± standard error.

## RESULTS AND DISCUSSIONS

### Isolation and Preliminary Screening on Polyunsaturated Fatty Acid (PUFA)

Six species were isolated from Teluk Aling, Penang, that might be considered as good aquaculture candidates, which include *Isochrysis maritima* (length: 3–7 μm), *Isochrysis galbana* (length: 1.5–10 μm), *Chaetoceros calcitrans* (length: 3–9 μm), *Tetraselmis tetrathele* (length: 10–16 μm), *Chlorella sorokiniana* (length: 1.5–10 μm) and *Nannochloropsis oculata* (length: 1–2 μm) after incubation in Walne’s media without the addition of vitamins. Table 1 shows the concentration of PUFAs of the six microalgae species isolated. All five important PUFAs namely linoleic acid (LA), linolenic acid (ALA), docosahexanoic acid (DHA), arachidonic acid (AA) and eicosapentanoic acid (EPA) were present in *I. maritima.* Four PUFAs (LA, ALA, EPA, AA and LA, ALA, DHA, AA) were detected in *T. tetrathele* and *I. galbana*, respectively. Meanwhile, three types of PUFAs recorded in two green microalgae *N. oculata* and *C. sorokiniana* were LA, ALA and DHA. *C. calcitrans* contained only two types of PUFAs (LA and EPA).

LA was the most abundant PUFAs detected in all six studied species except for *C. sorokiniana* which has ALA as the abundant PUFAs. DHA was detected in most of the studied species except for *T. tetrathele* and *C. calcitrans*, with *I. maritima* has the highest concentration of DHA. However, EPA and AA were only present in *I. maritima, C. calcitrans, T. tetrathele* and *I. galbana*. *C. calcitrans* has the highest concentration of EPA while *T. tetrathele* showed the highest AA concentration.

*I. maritima* is Prymnesiophytes which is widely known to have high composition of PUFAs especially EPA and DHA (Tatsuzawa & Takizawa 1995; Brown 2002; Mansour *et al.* 2005; Hemaiswarya *et al.* 2010). It was extensively used in the aquaculture feed industries (Carvalho *et al.* 2006; Nalder *et al.* 2015) especially *Pavlova* sp. and *I. galbana* (Borowitzka 1997; Fidalgo *et al.* 1998; Brown 2002; Yoshioka *et al.* 2012). According to Brown *et al.* (1997), Prymnesiophytes were rich with one or both of DHA and EPA. In this present study, *I. maritima* recorded the best PUFAs composition by containing all the PUFAs of interest, thus this strain might be a promising candidate for aquaculture feed. *I. galbana* and *T. tetrathele* also showed good PUFAs composition, but without EPA and DHA, respectively. Compared to *I. maritima* and *I. galbana*, *T. tetrathele* was reported to have low to moderate levels of DHA or EPA (Brown *et al*. 1997). Despite the fact that *I. galbana* is very useful in aquaculture feed industries (Sanchez *et al.* 2000), however, since EPA and DHA is one of the major requirements in aquaculture feed industries (Bandarra *et al.* 2003; Martínez-Fernández *et al.* 2006; Liu *et al.* 2016), *I. maritima* has been chosen for further nutritional studies instead of *I. galbana*.

Amongst the studied species, *C. calcitrans* showed the most insignificant composition of PUFAs of interest. Although the EPA was present, however, with the absence of DHA, AA and also ALA, the strain can be considered not suitable for aquaculture feed. These findings were in agreement with Samsudin (1992) who reported a low content of PUFA in *C. calcitrans*. *T. tetrathele*, *N. oculata*, meanwhile, *C. sorokiniana* did not show significant composition of PUFAs of interest that are suitable to be applied in aquaculture (Table 1). Since ALA and LA can be converted to AA and EPA, their concentration should not be ignored. Based on our results, *I. maritima* showed the best PUFAs composition. Although the concentration was not high, but they contained all of the important PUFAs. With this distinguish features, *I. maritima* has been chosen to be carried out for further experiment.

### Growth Rate and Biochemical Compositions of *Isochrysis maritima* in Batch Cultivation Systems

**Figure 1:**
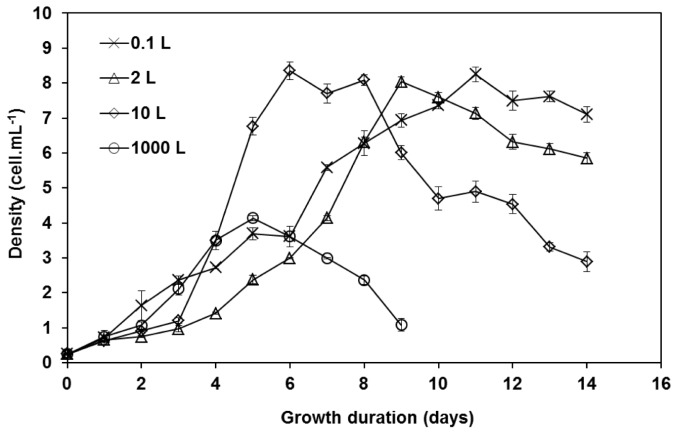
Growth curve of *I. maritima* in different volumes of Walne’s medium (mean density ± s.e.)

**Figure 2:**
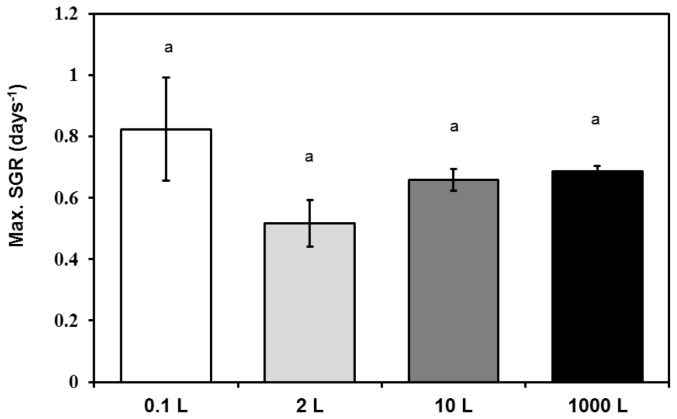
Maximum specific growth rate (SGR) of *I. maritima* in different volumes of Walne’s medium (mean ± s.e.)

*Isochrysis maritima* was cultivated through batch culture in 0.1 L, 2 L, 10 L and 1000 L Walne’s media. As shown in Figure 1, maximum cell densities of *I*. *maritima* grown in different volumes of Walne’s media were not significantly different (One-way ANOVA, *p* > 0.05), except for 1000 L which was significantly the lowest. *I. maritima* cultured in 0.1 L culture volume showed the fastest maximum specific growth rate (0.82 days^−1^), followed by 0.69 day^−1^ in 1000 L Walne’s medium. The slowest growth rate was 0.52 day^−1^, recorded in 2 L Walne’s medium, meanwhile in 10 L medium, the specific growth rate was 0.66 day^−1^ (Fig. 2). However, there were no significant difference between them statistically (One-way ANOVA, *p* > 0.05).

Based on the results obtained, *I. maritima* fit the attributes listed by Brown (2002) for microalgae to be considered as useful aquaculture species, which include rapid growth rates, applicable for mass cultivation and stable to any fluctuation in culture conditions. The maximum specific growth rate of this species was fast (between 0.52–0.82 day^−1^ depending on the size of the culture media and culture conditions), which was comparable to other aquaculture species such as *Nannochloropsis* sp. (0.23 day^−1^) (Richmond & Cheng-wu 2001), *Isochrysis galbana* (0.62 day^−1^) (Zhu *et al.* 1997), *Nannochloris oculata* (0.42 day^−1^) (Cho *et al.* 2007) and *Rhodomonas* sp. (0.35 day^−1^) (Renaud *et al*. 2002). On the other hand, this species was also able to be cultivated successfully in big volume (1000 L tank) under open hatchery condition exposed to uncontrolled environmental condition, which will optimise the production cost without relying to any high technology equipment.

**Figure 3:**
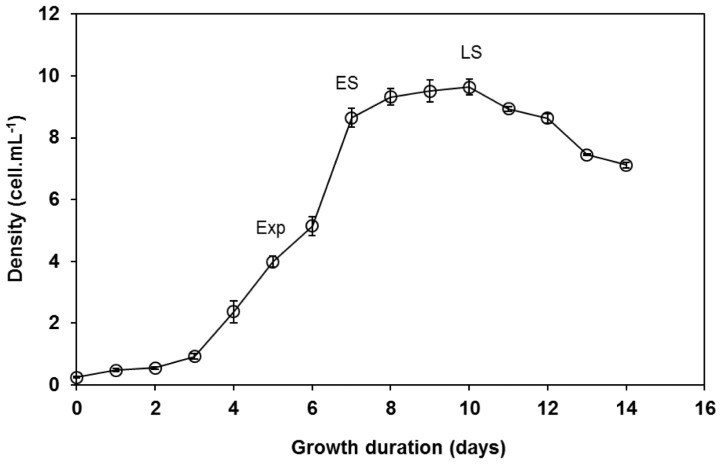
Growth curve of *I. maritima* in 10 L Walne’s media. Labels indicate harvesting points: Exponential (Exp), Early stationary (ES), Late stationary (LS)

In order to fulfil other attributes listed by Brown (2002), where microalgae should possess good nutrient composition, the total protein, total carbohydrate and fatty acid composition of *I. maritima* were measured at different growth phases which were determined by growth curve plotted as showed in Figure 3. Table 2 shows that protein content was affected by the growth phases. The protein content significantly decreased as the culture aged from exponential phase (21.4±4.8% AFDW) to late stationary phase (10.4±1.56% AFDW). Meanwhile, the carbohydrate content also decreased from exponential phase (32.1 ± 6.22 % AFDW) to late stationary phase (19.7±2.44% AFDW) but statistical analysis proved that there were no significant differences among them (*p* > 0.05). The protein and carbohydrate accumulation in photosynthetic microalgae was closely related with their cell metabolism. It was widely reported that the biochemical composition of rapidly growing cells at exponential phase is generally be marked by a high protein content to support cell growth and cell division (Dortch *et al*. 1984; Utting 1985) and low carbohydrate content. However, under growth limiting conditions, more photo assimilated carbon is fused into carbohydrate and lipids or both, due to low availability of nitrogen (Fidalgo *et al.* 1995), which led to high carbohydrate content at stationary growth phase (Zhu *et al.* 1997; Phatarpekar *et al*. 2000).

The fatty acids composition of *I. maritima* at different growth phases is listed in Table 3. The main fatty acids in *I. maritima* (as percent of total fatty acids) were C14: 0 (13.74% – 20.35%) and C16 : 0 (15.14% – 21.72%) from saturated fatty acids (SFA), C18 : 1 (n–9) (3.81% – 5.59%) from monosaturated fatty acids (MUFA) together with C18 : 2 (n-6) (2.32 – 4.04%) and C22 : 6 (n–3) (7.38% – 9.28%) from polyunsaturated fatty acids (PUFA). The *I. maritima* harvested at late stationary phase showed the highest SFA content which recorded 40.93 ± 3.21% of total fatty acids, followed by at early stationary phase and exponential phase which recorded 36.76±3.02% and 38.67±3.10%, respectively, but there were no significant differences exist between them (One-way ANOVA, *p* > 0.05). The similar results were also obtained for MUFA & PUFA with no significant differences among the growth phases (*p* < 0.05).

The results obtained showed that *I. maritima* possess a good nutrient composition especially omega-3 PUFA which is essential diet for many commercial aquaculture species, and harvesting the biomass at early stationary growth phase may allow better yield in term of total protein, total carbohydrate and omega 3 PUFA. Besides, low ratio of omega-6 to omega-3 recorded regardless of growth phases proved that this species is suitable for feeding. These ratios are used in bivalve nutrition which lower values of omega-6 to omega-3 (lower than 0.5) indicate optimal value for larvae and juvenile oysters feeding (Fidalgo *et al*. 1998).

## CONCLUSION

Being one of the Prymnesiophyceae, *I*. *maritima* was less reported compared to *I. galbana* and *Pavlova* sp. which are popular choice for aquaculture feed. Our study showed that this species is also a suitable candidate for aquaculture, which meets all the general attributes, including appropriate size for ingestion, rapid growth rates, amenable for mass culture, stable to any fluctuation in culture conditions and also have good nutrient composition.

## Figures and Tables

**Table 1:** Concentration (% area) of polyunsaturated fatty acids (PUFAs) in six species of microalgae

Microalgae	LA	ALA	DHA	EPA	AA
Isochrysis maritima	31.64	18.63	6.74	0.45	0.16
Tetraselmies tetrathele	33.82	14.80	–	0.34	2.24
Chaetoceros calcitrans	0.39	–	–	1.76	–
Isochrysis galbana	21.18	7.36	6.54	–	0.39
Nannochloropsis oculata	60.38	7.66	0.11	–	–
Chlorella sorokiniana	19.62	19.94	0.16	–	–

LA: linoleic acid; ALA: linolenic acid; DHA: docosahexanoic acid; AA: arachidonic acid; EPA: eicosapentanoic acid; -: no detected

**Table 2:** Cell density, dry weight (DW), ash content, ash-free dry weight (AFDW), protein and carbohydrate content of *I. maritima* harvested at different growth phases

Parameters	Exp	ES	LS
Density (x 10^6^ cell.mL^−1^)	8.65±0.30^a^	9.64±0.25^b^	8.63±0.15^a^
DW (g.L^−1^)	3.14±0.11^a^	3.50±0.12^b^	3.13±0.07^a^
Ash (% DW)	22.9±1.27^a,b^	24.5±0.57^b^	21.4±0.31^a^
AFDW (g.L^−1^)	2.42±0.04^a^	2.64±0.1^a^	2.46±0.06^a^
Protein (% AFDW)	21.4±4.8^b^	20.6±0.34^b^	10.4±1.56^a^
Carbohydrate (% AFDW)	32.1±6.22^a^	31.5±5.09^a^	19.7±2.44^a^

The values presented are means of three replicates and standard errors. Means were compared using the multiple range test of Duncan (α = 0.05); differences were not significant for groups with the same letter.

Exp: Exponential phase; ES: early stationary phase; LS: late stationary phase.

**Table 3:** Fatty acids composition (% of total fatty acids) of *I. maritima* at different growth phases

Fatty acids	Exp	ES	LS
Saturated (SFA)			
C14 : 0	15.89±2.15	16.02±3.38	17.24±3.11
C15 : 0	1.15±0.24	1.34±0.31	2.12±0.21
C16 : 0	17.90±2.76	18.88±2.84	19.20±2.52
C17 : 0	0.56±0.08	0.35 ±0.06	0.78±0.13
C18 : 0	0.74±0.03	1.52±0.28	0.96±0.08
C20 : 0	0.28±0.06	0.21±0.06	0.35±0.12
C22 : 0	0.24±0.06	0.35±0.10	0.28±0.03

Monounsaturated (MUFA)			
C15 : 1	0.26±0.04	-	0.18±0.08
C16 : 1	1.27±0.31	1.10±0.05	1.22±0.14
C17: 1	0.16±0.05	-	0.06±0.06
C18 : 1 (n-9)	4.62±0.24	4.39±0.58	5.38±0.21
C18 : 1(n-7)	1.30±0.14	-	1.10±0.14

Polyunsaturated (PUFA)			
C18 : 2(n-6)	2.59±0.27	2.41±0.03	3.89±0.15
C18: 3(n-6)	-	0.08±0.02	-
C18 : 3 (n-3)	1.21±0.06	1.11±0.14	1.21±0.06
C20 : 3 (n-6)	0.07±0.02	-	0.02±0.01
C20 : 4 (n-6)	0.42±0.06	0.66±0.13	0.61±0.04
C20 : 5 (n-3)	-	0.3±0.18	-
C22 : 6 (n-3)	7.90±0.52	8.30±0.92	8.90±0.38

∑SFA	36.76±3.02^a^	38.67±3.10^a^	40.93±3.21^a^
∑MUFA	7.61±0.81^a^	5.49±1.65^a^	7.94±0.98^a^
∑PUFA	12.19±1.43^a^	12.86±1.28^a^	14.63±1.63^a^
∑ (n-3)	9.11±3.35^a^	9.71±2.54^a^	10.11±3.85^a^
∑ (n-6)	3.08±0.79^a^	3.15±0.70^a^	4.52±1.20^a^
∑(n-6)(n-3)	0.34	0.32	0.45

The values presented are means of three replicates. Means were compared using the multiple range test of Duncan (tukar symbol alpha = 0.05), differences were not significant for groups with the same letter.

Exp: Exponential phase; ES: early stationary phase; LS: late stationary phase; -: not detected

## References

[b1-tlsr-28-2-163] Abel K, Deschmertzing H, Peterson JI (1963). Classification of microorganisms by analysis of chemical composition. Journal of Bacteriology.

[b2-tlsr-28-2-163] Alkhamis Y, Qin JG (2013). Cultivation of Isochrysis galbana in Phototrophic, Heterotrophic, and Mixotrophic conditions. BioMed Research International.

[b3-tlsr-28-2-163] Andersen RA, Berges JA, Harrison PJ, Watanabe MM, Andersen RA (2005). Appendix A-Recipes for freshwater and seawater media. Algal Culturing Techniques.

[b4-tlsr-28-2-163] Bandarra NM, Pereira PA, Batista I, Vilela MH (2003). Fatty acids, sterols and α-tocopherol in *Isochrysis galbana*. Journal of Food Lipids.

[b5-tlsr-28-2-163] Borowitzka MA (1997). Microalgae for aquaculture: Opportunities and constraints. Journal of Applied Phycology.

[b6-tlsr-28-2-163] Bransden MP, Butterfield GM, Walden J, McEvoy LA, Bell JG (2005). Tank colour and dietary arachidonic acid affects pigmentation, eicosanoid production and tissue fatty acid profile of larval Atlantic cod (*Gadus morhua*). Aquaculture.

[b7-tlsr-28-2-163] Brown MR, Jeffrey SW, Volkman JK, Dunstan GA (1997). Nutritional properties of microalgae for mariculture. Aquaculture.

[b8-tlsr-28-2-163] Brown MR, Cruz-Suarez LE, Ricque-Marie D, Tapia-Salazar M, Gaxiola-Cortes MG, Simoes N (2002). Nutritional value of microalgae for aquaculture.

[b9-tlsr-28-2-163] Carvalho AP, Pontes I, Gaspar H, Malcata FX (2006). Metabolic relationship between macro- and micronutrients, and the eicosapentaenoic acid and docosahexanoic acid contents of *Pavlova lutheri*. Enzyme and Microbial Technology.

[b10-tlsr-28-2-163] Cho SH, Ji SC, Hur SB, Bae J, Park I-S, Song YC (2007). Optimum temperature and salinity conditions for growth of green algae *Chlorella ellipsoidea* and *Nannochloris oculata*. Fisheries Science.

[b11-tlsr-28-2-163] Chu WL, Phang SM, Goh SH (1996). Environmental effects on growth and biochemical composition of *Nitzschia inconspicua* Grunow. Journal of Applied Phycology.

[b12-tlsr-28-2-163] Dortch Q, Clayton JR, Thoresen SS, Ahmed SI (1984). Species differences in accumulation of nitrogen pools in phytoplankton. Marine Biology.

[b13-tlsr-28-2-163] DuBois M, Gilles KA, Hamilton JK, Rebers PA, Smith F (1956). Colorimetric method for determination of sugars and related substances. Analytical Chemistry.

[b14-tlsr-28-2-163] Fidalgo JP, Abalde ACJ, Gerrero C (1995). Culture of the marine diatom *Phaeodactylum tricornutum* with different nitrogen sources: Growth, nutrient conversion and biochemical composition. Cahiers de Biologie Marine.

[b15-tlsr-28-2-163] Fidalgo JP, Cid A, Torres E, Sukenik A, Herrero C (1998). Effects of nitrogen source and growth phase on proximate biochemical composition, lipid classes and fatty acid profile of the marine microalga *Isochrysis galbana*. Aquaculture.

[b16-tlsr-28-2-163] Glencross BD (2009). Exploring the nutritional demand for essential fatty acids by aquaculture species. Reviews in Aquaculture.

[b17-tlsr-28-2-163] Guedes AC, Malcata FX, Muchlisin ZA (2012). Nutritional value and uses of microalgae in aquaculture. Aquaculture.

[b18-tlsr-28-2-163] Helm MM, Bourne N, Lovatelli A (2004). Hatchery culture of bivalves: A practical manual.

[b19-tlsr-28-2-163] Hemaiswarya S, Raja R, Ravi Kumar R, Ganesan V, Anbazhagan C (2010). Microalgae: A sustainable feed source for aquaculture. World Journal of Microbiology and Biotechnology.

[b20-tlsr-28-2-163] Kawachi M, Noël MH, Andersen RA (2005). Chapter 5: Sterilization and sterile technique. Algal Culturing Techniques.

[b21-tlsr-28-2-163] Liu W, Pearce CM, Mckinley RS, Forster IP (2016). Nutritional value of selected species of microalgae for larvae and early post-set juveniles of the Pacific geoduck clam, *Panopea generosa*. Aquaculture.

[b22-tlsr-28-2-163] Lowry OH, Rosebrough NJ, Farr AL, Randall RJ (1951). Protein measurement with the Folin phenol reagent. Journal of Biological Chemistry.

[b23-tlsr-28-2-163] Mansour MP, Frampton DMF, Nichols PD, Volkman JK, Blackburn SI (2005). Lipid and fatty acid yield of nine stationary-phase microalgae: Applications and unusual C24–C28 polyunsaturated fatty acids. Journal of Applied Phycology.

[b24-tlsr-28-2-163] Martínez-Fernández E, Acosta-Salmón H, Southgate PC (2006). The nutritional value of seven species of tropical microalgae for black-lip pearl oyster (*Pinctada margaritifera*, L.) larvae. Aquaculture.

[b25-tlsr-28-2-163] Mata TM, Martins AA, Caetano NS (2010). Microalgae for biodiesel production and other applications: A review. Renewable and Sustainable Energy Reviews.

[b26-tlsr-28-2-163] Muller-Feuga A, Richmond A (2004). Microalgae for aquaculture The current global situation and future trends. Handbook of Microagal Culture.

[b27-tlsr-28-2-163] Nalder TD, Miller MR, Packer MA (2015). Changes in lipid class content and composition of *Isochrysis* sp. (T-Iso) grown in batch culture. Aquaculture International.

[b28-tlsr-28-2-163] Pahl SL, Lewis DM, Chen F, King KD (2010). Heterotrophic growth and nutritional aspects of the diatom *Cyclotella cryptica* (Bacillariophyceae): Effect of some environmental factors. Journal of Bioscience and Bioengineering.

[b29-tlsr-28-2-163] Patil V, Reitan KI, Knudsen G, Mortensen L, Kallqvist T, Olsen E (2005). Microalgae as source of polyunsaturated fatty acids for aquaculture. Current Topics in Plant Biology.

[b30-tlsr-28-2-163] Phatarpekar P, Sreepada R, Pednekar C, Achuthankutty C (2000). A comparative study on growth performance and biochemical composition of mixed culture of *Isochrysis galbana* and *Chaetoceros calcitrans* with monocultures. Aquaculture.

[b31-tlsr-28-2-163] Rausch T (1981). The estimation of micro-algal protein content and its meaning to the evaluation of algal biomass I.: Comparison of methods for extracting protein. Hydrobiologia.

[b32-tlsr-28-2-163] Reitan KI, Rainuzzo JR, Olsen Y (1994). Effect of nutrient limitation on fatty acid and lipid content of marine microalgae. Journal of Phycology.

[b33-tlsr-28-2-163] Renaud SM, Thinh LV, Lambrinidis G, Parry DL (2002). Effect of temperature on growth, chemical composition and fatty acid composition of tropical Australian microalgae grown in batch cultures. Aquaculture.

[b34-tlsr-28-2-163] Richmond A, Cheng-wu Z (2001). Optimization of a flat plate glass reactor for mass production of *Nannochloropsis* sp. outdoors. Journal of Biotechnology.

[b35-tlsr-28-2-163] Samsudin L (1992). Lipid and fatty acid composition of microalgae used in Malaysian aquaculture as live food for the early stage of penaeid larvae. Journal of Applied Phycology.

[b36-tlsr-28-2-163] Sanchez S, Martýnez ME, Espinola F (2000). Biomass production and biochemical variability of the marine microalga *Isochrysis galbana* in relation to culture medium. Biochemical Engineering Journal.

[b37-tlsr-28-2-163] Şirin S, Trobajo R, Ibanez C, Salvadó J (2011). Harvesting the microalgae *Phaeodactylum tricornutum* with polyaluminum chloride, aluminium sulphate, chitosan and alkalinity-induced flocculation. Journal of Applied Phycology.

[b38-tlsr-28-2-163] Spolaore P, Joannis-Cassan C, Duran E, Isambert A (2006). Commercial applications of microalgae. Journal of Bioscience and Bioengineering.

[b39-tlsr-28-2-163] Tatsuzawa H, Takizawa E (1995). Changes in lipid and fatty acid composition of *Pavlova lutheri*. Phytochemistry.

[b40-tlsr-28-2-163] Utting SD (1985). Influence of nitrogen availability on the biochemical composition of three unicellular algae of commercial importance. Aquacultural Engineering.

[b41-tlsr-28-2-163] Van Wychen S, Laurens LML (2013). Determination of Total Solids and Ash in Algal Biomass - Laboratory Analytical Procedure ( LAP ).

[b42-tlsr-28-2-163] Yoshioka M, Yago T, Yoshie-Stark Y, Arakawa H, Morinaga T (2012). Effect of high frequency of intermittent light on the growth and fatty acid profile of *Isochrysis galbana*. Aquaculture.

[b43-tlsr-28-2-163] Zhu CJ, Lee YK, Chao TM (1997). Effects of temperature and growth phase on lipid and biochemical composition of *Isochrysis galbana* TK1. Journal of Applied Phycology.

